# Polymerase chain reaction allelotyping of human ovarian cancer.

**DOI:** 10.1038/bjc.1994.79

**Published:** 1994-03

**Authors:** R. J. Osborne, V. Leech

**Affiliations:** Cancer Research Campaign Department of Clinical Oncology, University of Cambridge School of Clinical Medicine, Addenbrooke's Hospital, UK.

## Abstract

**Images:**


 © Macmillan Press Ltd., 1994

### Polymerase chain reaction allelotyping of human ovarian cancer

R.J. Osborne & V. Leech

*Cancer Research Campaign Department of Clinical Oncology, University of Cambridge School of Clinical Medicine, Addenbrooke's Hospital, Cambridge CB2 2QQ, UK.*

**Summary** We have used a set of microsatellite polymorphisms (MSPs) to examine the location and frequency of allele loss throughout the genome in a panel of 25 human epithelial ovarian tumours. When more than one MSP was employed per arm, mean informativity was 85.2% (range 64-100%). The average fractional allelic loss was 0.28 (range 0-0.65). A high frequency of allele loss was seen at 5q (40%), 9q (48%), 11p (43%), 14q (46%), 15q (40%), 17p (61%), 17q (64%), 19p (45%) and Xp (40%), confirming previous findings at some sites, but also suggesting the existence of new tumour-suppressor genes in regions (9q, 14q, 15q) which have not previously been studied in ovarian cancer. For 9q and 14q, partial loss of the arm was more common than loss of heterozygosity for all loci. There was a significant relationship between allele loss affecting the short arm of chromosome 17 and allele loss affecting 17q ($P < 0.001$). No other relationship was detected between allele losses at different sites. Polymerase chain reaction allelotyping is suitable for the examination of very small tumour samples and tumours in which classical karyotyping is problematic.

In the presence of a mutated tumour-suppressor gene, loss of the normal homologue unmasks the defective gene and allows unopposed dysfunction. A variety of mechanisms, including whole homologue loss, mitotic recombination and deletion, may result in loss of the normal gene. These varied phenomena may be manifested by loss of heterozygosity (LOH) at one allele of a heterozygous locus. The term 'deletion' is often used where LOH is observed, regardless of the underlying mechanism. In ovarian cancer, several chromosome regions (3p, 6p, 6q, 11p, 11q, 13q, 17p, 17q, Xp) have been reported to be frequently affected by allele loss (Ehlen & Dubeau, 1990; Okamoto *et al.*, 1991; Zheng *et al.*, 1991; Eccles *et al.*, 1992; Gallion *et al.*, 1992; Jones & Nakamura, 1992; Saito *et al.*, 1992; Viel *et al.*, 1992; Yang-Feng *et al.*, 1992; Jacobs *et al.*, 1993; Foulkes *et al.*, 1993*a*, *b*). In most sites, the genes involved are not yet characterised, though the high rate of deletion implies the presence of tumour-suppressor genes of considerable importance.

Studies of tumour progression in colonic neoplasia (Vogelstein *et al.*, 1988) suggest that the accumulation of genetic lesions may occur in a relatively consistent and ordered manner, with correlations between particular lesions and phenotypic and clinical parameters. In ovarian cancer, individual studies which have defined frequently deleted regions have also included assessments of clinical or pathological relationships (Zheng *et al.*, 1991; Gallion *et al.*, 1992; Viel *et al.*, 1992; Foulkes *et al.*, 1993*a*). However, because of the wide range of lesions which occur, this approach has not provided a clear insight into the disease process. Previous studies of limited numbers of regions have also failed to assess the total number of genetic lesions, another important factor in tumour phenotype (Vogelstein *et al.*, 1988).

Ideally, analysis of all relevant loci is required for a valid assessment of the relationship between genotype and phenotype. For tumour-suppressor genes (known and putative) this can be achieved in two ways: by direct visualisation of chromosomes and by allele loss studies which involve every arm of every chromosome ('allelotyping'). Although conventional karyotyping has provided pointers to regions where deletions are frequent (Whang-Peng *et al.*, 1984; Pejovic *et al.*, 1989), it has not been applied to sufficient tumours for conclusions to be drawn about tumor progression or other clinical features. Allelotyping using restriction fragment length polymorphisms (RFLPs) (Sato *et al.*, 1991; Cliby *et al.*, 1993) is limited by the low informativity of many loci, the limited number of RFLPs available and the requirement for relatively large amounts of tumour DNA. The recent development of large numbers of highly informative, well-distributed microsatellite polymorphisms (MSPs) (Todd, 1992) may allow a more comprehensive allelotype to be rapidly performed, using very small samples if necessary. We have used MSPs spanning every arm of every chromosome (excluding the short arms of the acrocentric chromosomes) to examine 25 paired ovarian tumour-blood lymphocyte DNA samples. We report on the feasibility of this approach, and the abnormalities detected.

Correspondence: R.J. Osborne
Received 19 August 1993; and in revised form 13 October 1993.

### Materials and methods

### *Tumours*

Twenty-five malignant epithelial ovarian tumours were studied. Samples comprised either surgically resected solid masses or ascites cells. Tumour masses were frozen at − 70°C before use. Ascites cells and lymphocytes were processed

**Table I** Patient details: tumour histology, grade, stage and origin

| *Tumour no.* | *FIGO stage* | *Histology* | *Grade* | *Tumour origin* |
|---|---|---|---|---|
| 3 | III | Serous | WD | Primary |
| 8 | IV | Serous | NS | Ascites |
| 10 | III | Serous | NS | Ascites |
| 11 | II | Serous | PD | Primary |
| 12 | I | Serous | MD | Ascites |
| 13 | III | Serous | PD | Primary |
| 16 | III | Adenocarcinoma | PD | Primary |
| 19 | III | Serous | PD | Primary |
| 20 | I | Serous | NS | Primary |
| 21 | IV | Serous | PD | Primary |
| 23 | III | Mucinous | MD | Ascites |
| 39 | II | Serous | PD | Primary |
| 40 | III | Clear cell | MD | Primary |
| 41 | NS | Adenocarcinoma | PD | Primary |
| 44 | III | Endometroid | PD | Primary |
| 45 | III | Serous | PD | Ascites |
| 47 | III | Serous | NS | Primary |
| 48 | III | Mucinous | MD | Primary |
| 49 | III | Adenocarcinoma | PD | Primary |
| 51 | III | Serous | PD | Primary |
| 52 | III | Adenocarcinoma | PD | Ascites |
| 54 | III | Endometroid | PD | Ascites |
| 55 | III | Adenocarcinoma | PD | Ascites |
| 56 | III | Serous | MD | Ascites |
| 62 | III | Serous | NS | Ascites |

Abbreviations: WD, well differentiated. MD, moderately differentiated. PD, poorly differentiated. NS, not specified.

**Table II** Microsatellite polymorphisms: identity, location and primer sequences

| *Chromosome arm* | *D number* | *Location* | *Reference, sequence* | *Reference, locus* | *Sequence* |
|---|---|---|---|---|---|
| 1p | AMY2B | 1p21 | G 1990-7-97 | PNAS 1983-80-6932 | AAA CCT CTG GCA GTG TAC AC<br>TAT TTA CTG TCC TTA TTT ATG TGG G |
| 1q | APOA2 | 1q21–q23 | AJHG 1989-44-388 | AJHG 1989-44-388 | CTGGATACCTTTTTGGGGAGG<br>TTGCCCTGAGACTTACTTGGC |
| 1q | D1S103 | 1q32–q44 | NAR 1990-18-2199 | S 1992-258-67 | ACGAACATTCTACAAGTTAC<br>TTTCAGAGAAACTGACCTGT |
| 2p | TPO | 2p23–pter | HMG 1992-1-137 | HMG 1992-1-137 | CAC TAG CACCCA GAA CCG TC<br>CCT TGT CAG CGT TTA TTT GCC |
| 2p | CD8A | 2p12 | NAR 1991-19-1718 | I 1989-30-393 | ACTGCCTCATCCAGTTTCAG<br>GAGCAGGCACTTGTTAGATG |
| 2q | D2S72 | 2q | NAR 1990-18-2200 | S 1992-258-67 | AGC TAT AAT TGC ATC ATT GCA<br>TGG TCT ATA ACT GGT CTA TG |
| 3q | ACPP | 3q21–qter | NAR 1991-19-4792 | CCG 1989-52-68 | GGGCAACATGGTGAAACCTT<br>CCTAGCCTATACTTCCTTTC |
| 3q | D3S196 | 3q | NAR 1990-18-4635 | S 1992-258-67 | ACT CTT TGT TGA ATT CCC AT<br>TTT CCA CTG GGG AAC ATG GT |
| 4p | D4S174 | 4p11–p15 | NAR 1990-18-4636 | G 1992-14-209 | AAGAACCATGCGATACGACT<br>CATTCCTAGATGGGTAAAGC |
| 4p | GABARB1 | 4p12–p13 | AJHG 1991-49-621 | AJHG 1991-49-621 | GCTCATTAAACACTGTGTTCCT<br>TGATAGCTAGAAAGCTAGCAAG |
| 4q | D4S175 | 4q25–34 | G 1992-14-209 | G 1992-14-209 | ATC TCT GTT CCC TCC CTG TT<br>CTT ATT GGC CTT GAA GGT AG |
| 4q | D4S171 | 4q35–qter | NAR 1990-18-2202 | G 1992-14-209 | TGGGTAAAGAGTGAGGCTG<br>GGTCCAGTAAGAGGACAGT |
| 5p | D5S268 | 5p | NAR 1991-19-5794 | NAR 1991-19-5794 | AAGGTGAGGCAAAATGAGTGTA<br>CAATCAGGCCATTTTTAACTTCA |
| 5p | D5S117 | 5p15.1–15.3 | NAR 1990-18-4035 | CCG 1991-58-284 | TGTCTCCTGCTGAGAATAG<br>TAATATCCAAACCACAAAGGT |
| 5q | D5S346 | 5q21–q22 | NAR 1991-19-6348 | AJHG 1988-43-638 | ACTCACTCTAGTGATAAATCGGG<br>AGCAGATAAGACAAGTATTACTAGTT |
| 6p | F13A1 | 6p24–p25 | NAR 1991-19-4306 | CCG 1988-48-25 | GAGGTTGCACTCCAGCCTTT<br>ATGCCATGCAGATTAGAAA |
| 6p | D6S109 | 6p21.3–p24 | NAR 1991-19-1171 | S 1992-258-67 | CACCCTGGGCAATAAGAGCG<br>CCCCTCTTCATCCTCCCTTTCA |
| 6p | FTHP1 | 6p12–p21.3 | NAR 1991-19-6969 | CCG 1985-40-696 | ATCAATGGAAAAATGGGTAA<br>TATCTTTCTCTGTCTGCCTT |
| 6q | D6S87 | 6q23.1 | NAR 1990-18-4636 | CCG 1991-58-323 | ACAGAGTGAGACCGTGTAAC<br>AGAGAAGCATCTCACTTAGT |
| 7p | EGFR | 7p11.2–p12 | HMG 1992-1-135 | G 1991-11-737 | GTT TGA AGA ATT TGA GCC AAC C<br>TTC TTC TGC ACA CTT GGC AC |
| 7q | D7S23 | 7q31 | NAR 1991-19-5798 | S 1989-245-1059 | GAC GTG CTA GCC TGG TCT CCA GCT CT<br>GAT GGG GGA GGC GGT TGT AGT TTT CAA |
| 7q | CFTR | 7q31 | AJHG 1991-49-1256 | S 1989-245-1059 | GCT GCA TTC TAT AGG TTA TC<br>TGT GAA AAC AGG GAT AAT AC |
| 8p | LPL | 8p22 | CCG 1991-58-1932 | CCG 1993-63-45 | ATC TGC CTC TGC AGC TCT CA<br>ATT CTG GTA TGA ATG TAC ATG TG |
| 8p | D8S135 | 8p | NAR 1991-19-6664 | S 1992-258-67 | GCTAATCAGGGAATCACCCAA<br>AAATACCGAGACTCACACTATA |
| 8p | ANK1 | 8p11.1–p21.1 | NAR 1991-19-969 | N 1990-344-36 | CACAGCTTCAGAAGTCACAG<br>TCCCAGATCGCTCTACATGA |
| 8q | D8S161 | 8q22–qter | NAR 1991-19-5093 | NAR 1991-19-5093 | GATCAAGGAGCATCACATCT<br>TAACATGTCCCCTCATTTGG |
| 9p | D9S54 | 9p22–pter | G 1992-12-607 | G 1992-12-607 | GAA AGT CCA GAA CTA AGT AG<br>TGT GGA TAG GTA TAT ATA GC |
| 9q | D9S15 | 9q13–q21.1 | HG 1990-85-98 | G 1992-14-715 | TAA AGA TTG GGA GTC AAG TA<br>TTC ACT TGA TGG TGG TAA TC |
| 9q | GSN | 9q33 | NAR 1991-19-967 | G 1992-14-715 | CAG CCA GCT TTG GAG ACA AC<br>TCG CAA GCA TAT GAC TGT AA |
| 9q | ASS | 9q34.1 | NAR 1990-18-7472 | G 1992-14-715 | GGGAGCTATAAAAATGACCA<br>TTAGGTCCGAAAACACAAAG |
| 9q | D9S64 | 9q34–qter | G 1992-12-229 | G 1992-14-715 | GAA GGG CTC TTT ATT AAC TGA T<br>AAC CTG GGC GAC ACA GCA A |
| 10p | D10S89 | 10p11.2–pter | NAR 1990-18-4637 | G 1992-12-604 | AAC ACT AGT GAC ATT ATT TTC A<br>AGC TAG GCC TGA AGG CTT CT |
| 10p | D10S111 | 10p11.2–pter | G 1992-12-604 | G 1992-12-604 | CCA AAG TGC TGA ATT TCA GG<br>GAA AAG TCT TAG AAT TTT GCA G |
| 10p | D10S179 | 10p | NAR 1992-20-1431 | NAR 1992-20-1431 | GCCCACTTTCAGATTCCTGCT<br>GCAGGGAGAAGGACTATGCAT |
| 10q | D10S173 | 10q | G 1992-13-622 | S 1992-258-67 | GCT GAT TTT TCC TGC TGG TC<br>TGT TTC TGA AGC ATT TTC CTT G |
| 11p | D11S419 | 11p13–p15.1 | NAR 1990-18-4036 | S 1992-258-67 | AGG GCT TCC TGT CCA TCT A<br>CTC ATT TGA AGA CTG CAG CA |
| 11q | D11S534 | 11q13 | NAR 1991-19-4308 | NAR 1991-19-4308 | ATA TGG AAA CTC TCC GTA CT<br>GCA ACC ATG GAG AGT CTG GA |
| 11q | D11S836 | 11q | MFD 108 | S 1992-258-67 | GCC TCT GAA GTG GCT AAA TA<br>CCC CTC ACC ACA TCA CTT G |
| 12p | F8VWF | 12p12–pter | NAR 1990-18-4957 | S 1985-228-1401 | TGT ACC TAG TTA TCT ATC CTG<br>GTG ATG ATG ATG GAG ACA GAG |
| 12q | D12S60 | 12q | MFD109 | S 1992-258-67 | GAC ACA GAG AAG GCA AAT AG<br>TCC CAT ATC CTA TGT AGA AG |

**Table II** – *cont.*

| *Chromosome arm* | *D number* | *Location* | *Reference, sequence* | *Reference, locus* | *Sequences* |
|---|---|---|---|---|---|
| 13q | FLT-1 | 13q12 | NAR 1991-19-2803 | O 1990-5-519 | TTTGGCCGACAGTGGTGTAA<br>AGGACCAAACCATGTCTGTC |
| 13q | D13S115 | 13q | G 1992-13-622 | S1992-258-67 | TGT AAG GAG AGA GAG ATT TCG ACA<br>TCT TAG CTG CTG GTG GTG G |
| 14q | D14S34 | 14q | NAR1990-18-4638 | MFD 42 | GGC CTC AAA GAA TCC TAC AG<br>GAC ACG TAG TTG CTT ATT AC |
| 14q | D14S50 | 14q | G 1992-13-532 | S 1992-258-67 | ATG ATT CCA CAA GAT GGC AG<br>AAC ACC CCT AAT TCA CCA CT |
| 14q | D14S49 | 14q | G 1992-13-532 | S 1992-258-67 | TCT ACA AAA AGT CAG ATA CCT<br>GAA TCT TAA GTA GTT ATC CCT C |
| 14q | D14S51 | 14q | G 1992-13-532 | S 1992-258-67 | GAT TCT GCA CCC CTA AAT CC<br>ATG CTC AAT GAA CAG CCT GA |
| 14q | D14S48 | 14q | G 1992-13-532 | S 1992-258-67 | CAA AAC AGA GAA CAG AGT AG<br>CAT AAA AGG CTT ATT GGT TTG |
| 15q | FES | 15q26.1 | NAR 1991-19-4018 | CCG 1993-63-33 | GGA AGA TGG AGT GGC TGT TA<br>CTC CAG CCT GGC GAA AGA AT |
| 16p | D16S292 | 16p13 | G 1992-13-402 | G 1992-13-402 | GGCATGTCAGGCCAGCCATGTTTT<br>CTTTGCACAAAAACAGTAGCTATCCAC |
| 16q | D16S265 | 16q21 | NAR 1990-18-4034 | S 1992-258-67 | CCA GAC ATG GCA GTC TCT A<br>AGT CCT CTG TGC ACT TTG T |
| 17p | D17S520 | 17p12 | MFD 144 | CCG 1991-58-728 | GGA GAA AGT GAT ACA AGG GA<br>TAG TTA GAT TAA TAC CCA CC |
| 17p | TP53 | 17p13.1 | GCC 1992-5-89 | N 1986-320-84 | AGG GAT ACT ATT CAG CCC GAG GTG<br>ACT GCC ACT CCT TGC CCC ATT C |
| 17q | D17S250 | 17q11.2–q12 | NAR 1990-18-4640 | NAR 1990-18-4640 | GGA AGA ATC AAA TAG ACA AT<br>GCT GGC CAT ATA TAT ATT TAA ACC |
| 17q | D17S588 | 17q12–q21 | CR 1993-53-1218 | CR 1993-53-1218 | CCT GGT CTA GGA AGA GTG TCA<br>GTG TAA GCA TCT GTG TAT ACT AC |
| 18p | D18S40 | 18p11.21–pter | G 1993-15-48 | G 1993-15-48 | CAA GAT AGA TGC ATT TTC CAG T<br>CAT CCA AAG GGT GAA TGT GT |
| 18q | D18S35 | 18q21.2–21.3 | NAR 1990-18-6465 | G 1993-15-48 | AGC TAG ATT TTT ACT TCT CTG<br>CTG GTT GTA CAT GCC TGA C |
| 19p | D19S177 | 19p13.3 | CCG 1991-58-1190 | S 1992-258-67 | TCA GAG GTT GAG GCT GAA G<br>CAA TGA CTT CAA GCA CTA AG |
| 19q | D19S49 | 19q12 | NAR 1990-18-1927 | S 1992-258-67 | ACT CAT GAA GGT GAC AGT TC<br>GTG TTG TTG ACC TAT TGC AT |
| 20p | D20S27 | 20p12 | NAR 1990-18-2202 | S 1992-258-67 | TTT ATG CGA GCG TAT GGA TA<br>CAC CAC CAT TGA TCT GGA AG |
| 20q | D20S54 | 20q | G 1992-12-183 | G 1992-12-183 | TGA CCA GGT GTG ACA AGA TG<br>TTT AAC CTT TGG GAT TGT TTC |
| 20q | D20S46 | 20q | G 1992-12-183 | G 1992-12-183 | TAT GGT GGG AAG TCC AGC ATT G<br>AGG AGG AGG GAG ACC CCA GG |
| 21q | D21S120 | 21q11.2 | NAR 1990-18-4969 | NAR 1990-18-4969 | GTG TGT CTG CCA TTT CTG GGT GTA G<br>GAT CCT GGG ACA AAG TAG TCT CTA A |
| 21q | D21S171 | 21q22.3–qter | HG 1991-87-401 | HG 1991-87-401 | TAG GCC CTA CTG CAA TAA TG<br>CTT TAT CTT CAC ACA GCT TC |
| 21q | D21S167 | 21q22.3 | NAR 1990-18-4967 | NAR 1990-18-4967 | TCC TTC CAT GTA CTC TGC A<br>TGC CCT GAA GCA CAT GTG T |
| 22q | D22S156 | 22q | NAR 1990-18-4639 | S 1992-258-67 | AGC CTG GGA GTC AGA GTG A<br>AGC TCC AAA TCC AAA GAC GT |
| 22q | TOPIP2 | 22q11.2–13.1 | HMG 1992-1-6 | HG 1989-84-6 | GGT TTT CTG TCA TTC TTG TTG A<br>AGT GAG TGG AGA TTG CAT TG |
| Xp | DXS538 | Xp11.21–21.1 | NAR 1991-19-1161 | AJHG 1990-46-776 | CTG ATT CAC TGT ACA ATG GT<br>ATG GAT AAT AAA CAG ACA GGA |
| Xq | DXS454 | Xq | NAR 1990-18-4037 | S 1992-258-67 | AGA AGA CAT AAG GAT ACT GC<br>GAT CCC AAC TAT TTC TTT CT |

MSPs are listed by their official locus name ('D number'), chromosomal location and oligonucleotide primer sequences. References relate to the published details of the sequence and location of each MSP. Journal abbreviations are listed below.
Journal abbreviations: AJHG, *American Journal of Human Genetics*; C, *Cell*; CCG, *Cytogenetics and Cell Genetics*; CGC, *Cancer Genetics and Cytogenetics*; CR, *Cancer Research*; G, *Genomics*; GCC, *Genes, Chromosomes and Cancer*; HG, *Human Genetics*; HMG, *Human Molecular Genetics*; I, *Immunogenetics*; MFD, Marshfield Markers Release 10–7/1/93 (J. Weber, personal communication); N, *Nature*; NAR, *Nucleic Acids Research*; O, *Oncogene*; PNAS, *Proceedings of the National Academy of Sciences of the USA*; S, *Science*.

fresh. Histological type, grade, clinical stage and origin of the tumours are detailed in Table I.

*DNA preparation*

Ten micron haematoxylin and eosin-stained frozen sections were examined to identify regions of tumour which were free from significant contamination with normal tissue. Two to ten further 10 μm sections were cut, and where necessary normal tissue was scraped away. No tumour sample contained more than 40% normal tissue. The sections were digested with proteinase K at 55°C in polymerase chain reaction (PCR) buffer for 1 h then boiled for 10 min. The resulting solution was used directly in the PCR reaction without further purification. Cytospin examination of ascites cells was performed, and only samples comprising greater than 60% tumour were used. Cells from 1 ml of fluid were added to a buffer containing detergents which lysed cytoplasmic membranes (Higuchi, 1989). The nuclei were pelleted and washed, then the nuclear membranes were digested in 1 ml of PCR buffer. Normal DNA was derived from the lymphocytes in 1 ml of whole blood, treated in the same way as ascites. An aliquot of the resulting solution was used directly in the PCR reaction without further purification.

*Oligonucleotides*

Oligonucleotides were obtained from the HGMP Resource Centre (Harrow, UK), or were synthesised locally. They were selected on the basis of their high informativeness, accurate

**Table III** Results for all loci

| | | | | TUMOURS | | | | | | | | | | | | | | | | | | | | | | | | | LOCUS | ARM | ARM |
|---|---|---|---|---|---|---|---|---|---|---|---|---|---|---|---|---|---|---|---|---|---|---|---|---|---|---|---|---|---|---|---|
| | | | TUMOUR NUMBER--> | 3 | 8 | 10 | 11 | 12 | 13 | 16 | 19 | 20 | 21 | 23 | 39 | 40 | 41 | 44 | 45 | 47 | 48 | 49 | 51 | 52 | 54 | 55 | 56 | 62 | % LOH | % LOH | % INF |
| | Arm | Locus | Location | | | | | | | | | | | | | | | | | | | | | | | | | | | | |
| 1 | 1p | AMY2B | 1p21 | | o | o | o | | | o | | o | • | o | o | o | o | o | | • | | o | | o | o | | • | | 19 | 19 | 64 |
| | 1q * | APOA2 | 1q21-q23 | | | o | | o | o | | o | o | | o | | o | | • | o | o | o | o | o | • | • | o | • | o | 22 | 23 | 88 |
| | 1q | D1S103 | 1q32-q44 | o | | | • | | | o | | o | | o | o | o | | | o | o | o | o | o | o | o | | • | o | 13 | | |
| 2 | 2p | TPO | 2p23-pter | o | | o | | | | | o | | o | o | o | o | o | | o | o | • | | | | • | | o | o | 14 | 17 | 92 |
| | 2p | CD8A | 2p12 | o | • | | o | o | o | o | o | o | | o | o | o | o | • | o | o | • | o | | | | o | o | o | 15 | | |
| | 2q | D2S72 | 2q | o | • | o | • | | | | • | • | o | o | | | • | o | | o | | o | | o | • | | o | o | 38 | 38 | 64 |
| 3 | 3p # | THRB | 3p24 | | o | | • | • | o | • | o | o | o | | o | | o | • | | | o | o | o | | | o | o | • | 29 | | |
| | 3p # | D3S15S2 | 3p21 | o | | o | | | | • | o | o | o | o | | | o | o | | | | o | | | | o | | • | 17 | 26 | 92 |
| | 3p # | D3S30 | 3p13-p14 | | o | | | o | o | | o | | | o | | o | o | o | | o | o | o | | • | | o | | | 8 | | |
| | 3q * | ACPP | 3q21-qter | o | | | • | | o | | | | | o | o | | o | | | | • | o | | o | • | o | o | • | 31 | 32 | 88 |
| | 3q | D3S196 | 3q | o | o | • | • | o | | • | o | • | o | o | | o | | | o | | • | o | | o | o | o | o | o | 26 | | |
| 4 | 4p | D4S174 | 4p11-p15 | o | o | | | o | o | | • | o | | o | | o | o | • | o | o | o | o | o | • | o | o | o | o | 15 | 18 | 88 |
| | 4p * | GABRB1 | 4p12-p13 | o | o | o | • | o | o | | o | o | | o | | | | • | | o | | o | o | | o | o | | o | 13 | | |
| | 4q | D4S175 | 4p25-q34 | o | o | • | • | | | • | • | • | o | o | o | o | o | o | o | o | o | o | | • | | | o | • | 35 | 33 | 96 |
| | 4q | D4S171 | 4q35-qter | | | • | • | | o | | o | | o | | o | o | o | | | o | | o | o | • | • | o | o | o | 25 | | |
| 5 | 5p * | D5S268 | 5p | | • | | | o | o | • | • | o | | | o | o | | | o | o | | o | | | o | o | o | | 21 | 19 | 84 |
| | 5p | D5S117 | 5p15.1-15.3 | o | | | • | o | | | • | | | o | | o | o | o | | o | o | | | o | o | o | | | 15 | | |
| | 5q | D5S346 | 5q21-q22 | | o | | o | | o | | | | | o | | | • | • | o | o | | o | • | • | • | o | • | o | 40 | 40 | 60 |
| 6 | 6p | F13A1 | 6p24-25 | | • | | • | o | o | | o | o | | o | | o | o | • | | o | o | o | o | • | | o | o | o | 22 | | |
| | 6p * | D6S109 | 6p21.3-p24 | o | • | | • | o | o | | • | o | | | o | o | | o | o | | o | o | o | o | | | o | o | 18 | 27 | 88 |
| | 6p * | FTHP1 | 6p12-p21.3 | | | o | • | o | | | • | • | | | o | o | • | • | o | o | | o | | o | | | o | | 36 | | |
| | 6q | D6S87 | 6q23.1 | o | | o | • | o | o | | o | • | | o | | o | • | • | | o | o | | | • | • | o | o | | 35 | 35 | 68 |
| 7 | 7p | EGFR | 7p11.2-p12 | o | • | • | o | o | | | o | o | o | o | | | • | o | | o | | o | | o | o | o | o | o | 17 | 17 | 72 |
| | 7q | D7S23 | 7q31 | o | | | o | | | | o | | | o | | | • | | | o | | | | o | o | o | o | o | 9 | 19 | 64 |
| | 7q | CFTR | 7q31 | o | • | o | | | o | | | | | o | | | | • | o | | | | | | o | | o | | 22 | | |
| 8 | 8p | LPL | 8p22 | | | | o | o | | | | | | o | o | | o | • | | | | | | | | | | • | 29 | | |
| | 8p | D8S135 | 8p | • | | o | | o | o | | | o | o | o | o | | | | | o | o | | | • | | | • | | 25 | 26 | 76 |
| | 8p | ANK1 | 8p11.2 | | | | | | | | o | | | o | | | o | | o | o | o | | | | | o | o | • | 11 | | |
| | 8q | D8S161 | 8q22-qter | • | • | o | o | | | | • | o | o | o | | • | o | o | o | o | o | | o | o | o | | • | o | 26 | 26 | 76 |
| 9 | 9p | D9S54 | 9p22-pter | o | | | • | | | | | o | | | | o | • | | o | | | | o | | | o | • | o | 30 | 30 | 40 |
| | 9q | D9S15 | 9q13-q21.1 | o | • | o | • | | | o | • | o | | | | o | • | | | o | o | | | o | • | | o | | 36 | | |
| | 9q | GSN | 9q33 | | • | o | • | o | o | | • | | | o | | o | | • | • | o | o | o | • | • | | o | • | o | 39 | 48 | 100 |
| | 9q | ASS | 9q34.1 | o | • | | • | o | o | • | | | o | o | | o | | o | • | o | o | o | o | • | • | o | • | | 37 | | |
| | 9q | D9S64 | 9q34-qter | | | | • | | o | • | • | • | | o | o | o | • | o | • | o | o | | | • | | o | • | o | 47 | | |
| 10 | 10p | D10S89 | 10p11.2-pter | o | | o | o | | o | | | | | o | o | o | o | | | o | o | o | | o | | o | | o | 0 | | |
| | 10p | D10S111 | 10p11.2-pter | | | o | o | | | | | o | | o | o | o | o | | | o | | o | | | | o | | o | 0 | 22 | 92 |
| | 10p | D10S179 | 10p | | o | • | o | o | | | • | o | | o | o | | | • | o | | o | o | o | • | o | o | • | o | 28 | | |
| | 10q | D10S173 | 10q | o | | | o | o | | o | o | | | o | | o | • | o | • | o | o | o | | • | o | o | | | 19 | 19 | 64 |
| 11 | 11p | D11S419 | 11p13-p15.1 | | • | • | | o | o | • | • | | o | o | | o | o | • | | o | | o | | • | | | | | 43 | 43 | 56 |
| | 11q | D11S534 | 11q13 | | • | | | o | | | | o | o | | o | o | o | | | | | o | | • | • | o | • | o | 31 | 30 | 80 |
| | 11q | D11S836 | 11q | o | • | | | | | o | • | o | o | o | o | | o | • | o | o | | o | | • | • | | • | o | 35 | | |
| 12 | 12p* | F8VWF | 12p12-pter | o | | o | | o | o | o | | o | o | o | | | • | o | o | o | | | | o | | o | o | o | 6 | 6 | 64 |
| | 12q | D12S60 | 12q | | | | o | o | | • | | | o | o | o | | | o | o | o | o | o | | | • | o | o | o | 13 | 13 | 60 |
| 13 | 13q | FLT1 | 13q12 | | • | | | | o | o | | | | o | o | | | | | | | | | | | | | | 20 | 12 | 68 |
| | 13q | D13S115 | 13q | o | • | | | | o | | | | | o | o | | • | o | o | o | o | o | | o | o | o | o | o | 13 | | |
| 14 | 14q | D14S34 | 14q | | • | • | o | | | • | o | • | | | | | • | o | | o | | • | | | o | o | • | | 54 | | |
| | 14q | D14S50 | 14q | o | • | | o | o | o | | | | | o | | | | o | | | | | | • | | o | | • | 30 | | |
| | 14q | D14S49 | 14q | o | • | | o | o | o | • | • | o | | o | | o | o | o | o | o | o | o | | o | • | o | • | | 25 | 46 | 96 |
| | 14q | D14S51 | 14q | | | | o | o | o | • | | o | o | o | o | o | o | | o | o | o | | | • | o | | • | • | 24 | | |
| | 14q | D14S48 | 14q | | | | o | o | | | | • | | | o | o | | o | | o | | | | | | | • | • | 33 | | |
| 15 | 15q | FES | 15q26.1 | | | o | o | o | | o | | • | • | o | o | | | • | o | | • | o | | • | • | | | o | 40 | 40 | 60 |

localisation and even distribution. The loci and chromosmal regions examined and their corresponding oligonucleotides are detailed in Table II.

*PCR*

A 50–200 ng aliquot of genomic DNA (1 μl of solution described above) was amplified in a reaction volume of 12.5 μl as previously described (Jacobs *et al*., 1993). For all reactions except those indicated with an asterisk in Table II, PCR consisted of 1 min at 95°C, 2 min at 55°C and 2 min at 72°C for 30 cycles followed by a final extension for 10 min at 72°C. After chloroform extraction, PCR products were processed and analysed as previously described (Jacobs *et al*., 1993). For chromosome 3p an RFLP–PCR technique (Ganly *et al*., 1992) was used to examine five polymorphisms at the

**Table III** – *cont.*

| | | | | | | | | | | | | | | | | | | | | | | | | | | | | | | | |
|---|---|---|---|---|---|---|---|---|---|---|---|---|---|---|---|---|---|---|---|---|---|---|---|---|---|---|---|---|---|---|---|
| 16 | 16p | D16S292 | 16p13 | | | O | O | O | O | | O | O | O | O | O | O | O | O | | | | | | | | | | | 0 | 0 | 48 |
| | 16q | D16S265 | 16q21 | O | • | O | O | O | O | O | | • | | | O | O | O | O | O | | | O | | O | O | O | O | • | 16 | 16 | 76 |
| 17 | 17p | D17S520 | 17p12 | O | • | | | O | | O | • | • | O | O | | O | • | • | • | | | O | • | • | • | O | • | • | 58 | 61 | 92 |
| | 17p | TP53 | 17p13.1 | O | • | • | • | O | | • | | • | | O | O | | • | • | O | | | | • | • | • | O | • | • | 67 | | |
| | 17q | D17S250 | 17q11.2-q12 | O | • | • | • | O | • | • | • | • | O | O | O | O | • | • | • | O | • | O | • | • | • | | | | 64 | 64 | 88 |
| | 17q | D17S588 | 17q12-q21 | O | | • | • | | • | | | • | O | O | | | | • | | O | | O | | • | | | | | 55 | | |
| 18 | 18p | D18S40 | 18p11.21-pter | O | | O | | O | O | O | • | O | | | O | O | O | O | O | O | | O | | O | O | O | O | | 6 | 6 | 72 |
| | 18q | D18S35 | 18q21.2-q21.3 | O | | O | O | O | | O | | • | | O | | | | | O | O | • | O | O | O | | | • | • | 27 | 27 | 60 |
| 19 | 19p | D19S177 | 19p13.3 | O | | • | | | | | | O | O | | | O | O | • | • | O | | | | • | | | | • | 45 | 45 | 44 |
| | 19q | D19S49 | 19q12 | O | O | • | | | O | | | | O | O | | O | • | | O | | O | | | O | O | | • | | 23 | 23 | 52 |
| 20 | 20p | D20S27 | 20p12 | | | • | • | O | O | | O | | | O | O | | O | O | O | O | | | | O | | | • | O | 21 | 21 | 56 |
| | 20q* | D20S54 | 20q | O | O | | | O | O | | O | O | O | O | O | | O | O | | O | O | | | | O | | | • | 7 | 21 | 76 |
| | 20q* | D20S46 | 20q | O | O | • | • | O | O | | | O | | O | O | O | O | | | O | O | | | • | | | | | 21 | | |
| 21 | 21q | D21S120 | 21q11.2 | O | • | | • | | O | | | • | | O | O | | | O | O | | | | | O | | | O | • | 33 | | |
| | 21q | D21S171 | 21q22.3-qter | O | | | • | O | | O | O | • | O | O | | O | | | | O | | O | | • | O | | | | 23 | 33 | 84 |
| | 21q | D21S167 | 21q22.3 | | • | O | • | O | O | | • | • | | O | | | | | O | | | O | | • | | | • | • | 54 | | |
| 22 | 22q | D22S156 | 22q | O | • | • | | O | | O | O | O | | | | | | | | O | | O | | • | | | | | 30 | 28 | 72 |
| | 22q | TOPIP2 | 22q11.2-q13.1 | O | • | • | • | O | O | | O | O | | O | O | O | | | | O | | O | | • | O | O | | • | 29 | | |
| X | Xp | DXS538 | Xp11.21-p21.1 | O | | | O | | O | | | • | | O | O | O | | • | O | O | | | | • | • | • | • | O | 40 | 40 | 60 |
| | Xq | DXS454 | Xq | O | | | • | O | O | O | | | O | | O | O | O | • | O | O | O | | | | | | | • | 21 | 21 | 56 |

Allelotyping results for all tumours at all loci are presented. The symbols used are explained in the key below. The percentage loss of heterozygosity (LOH) at each locus and for each chromosomal arm has been calculated and is listed, with the percentage informativity of each locus, in the three columns at the right of the table.
Key: O, retained heterozygosity; ●, loss of heterozygosity; Blank, non-informative or failed. *Refer to original paper for PCR conditions; #RFLP–PCR (Osborne *et al.*, 1992).

THRB locus and two other proximal loci. Allele loss was assessed visually, and was scored when a clear reduction in intensity of one of the alleles was observed.

*Statistical analysis*

Fisher's exact test was used to determine whether a relationship existed between allele loss at different chromosomal sites.

### Results

PCR allelotyping of the tumour panel was relatively rapid and easy. Examination of a single locus, involving 50 samples plus controls, was completed within one working day (excluding autoradiography). Results for all tumours at all loci are shown in Table III. Table IV comprises a summary of these results analysed by chromosomal arm. Figure 1 summarises the percentage allele loss at each chromosomal arm. Informativeness for individual MSPs ranged between 20% and 88% (mean 59.1%). However, when more than one MSP was employed per arm, informativeness increased to a mean of 85.2% (range 64–100%).

Interpretation of results was facilitated by the use of tumours in which contamination with normal tissue had been minimised. The additional 'shadow' bands of smaller products routinely seen with this technique (Litt, 1991) did not hamper interpretation of results. Representative findings for one tumour–normal pair are shown in Figure 2. All results were scrutinised for evidence of microsatellite mutation (Thibodeau *et al.*, 1993). Only two examples were identified, each affecting a single locus (Figure 3).

Only two tumours (tumours 23 and 39) showed no evidence of deletion at any locus, whereas tumour 8 had allele loss affecting 65% of informative chromosomal arms. The mean allele loss per tumour was 28% (s.d. 22.8%). Because the material studied was derived predominantly from poorly differentiated serous stage III tumours, it was not possible to explore the relationship between frequency of allele loss and parameters such as tumour histology, grade or stage.

Frequency of loss of heterozygosity for individual chromosomal arms varied between 0% (16p) and 64% (17q). Forty per cent or more of informative tumours showed loss of heterozygosity at chromosomal arms 5q (40%), 9q (48%), 11p (43%), 14q (46%), 15q (40%), 17p (61%), 17q (64%), 19p (45%) or Xp (40%). There was a significant relationship between allele loss affecting the short arm of chromosome 17 and allele loss affecting 17q ($P < 0.001$). Non-disjunction is a possible explanation for this association. No other relationship was detected between allele losses at different sites in this cohort of tumours. Although allele loss usually affected all loci examined for a particular chromosomal arm, there were notable exceptions. For 9q and 14q partial loss of the arm was more common than loss of heterozygosity for all loci. This observation may explain the discrepancy between these results and those obtained in an earlier allelotyping study (Sato *et al.*, 1991) in which fewer loci were studied.

### Discussion

This paper describes the use of a set of microsatellite polymorphisms which permits a comprehensive evaluation of the numerous deletions which may occur throughout the genome of tumours. The MSPs selected are easy to use, particularly since the vast majority share common PCR conditions. The use of silver staining or automated sequencing techniques (Cawkwell *et al.*, 1993) to detect products are possible refinements which will further increase the utility of the method.

This approach depends upon the assumption that chromosome deletions are sufficiently large to allow their detection using probes which examine only a small number of loci per arm. Mapping studies employing large numbers of probes for a particular chromosome reveal that the majority of deletions are extensive, usually involving an entire arm (Jacobs *et al.*, 1993; Foulkes *et al.*, 1993*a*). Small interstitial or terminal deletions are relatively uncommon. In the present

**Table IV** Summary of results for individual chromosomal arms

| | | | | | | | | | | | T | U | M | O | U | R | S | | | | | | | | | | | |
|---|---|---|---|---|---|---|---|---|---|---|---|---|---|---|---|---|---|---|---|---|---|---|---|---|---|---|---|---|
| Chromosome | | 3 | 8 | 10 | 11 | 12 | 13 | 16 | 19 | 20 | 21 | 23 | 39 | 40 | 41 | 44 | 45 | 47 | 48 | 49 | 51 | 52 | 54 | 55 | 56 | 62 | % LOH | % INF |
| 1 | 1p | | o | o | o | | | o | | o | • | o | o | o | o | o | | • | | o | | o | o | | • | | **19** | 64 |
| | 1q | o | | o | • | o | o | o | o | o | | o | o | o | | • | o | o | o | o | o | • | • | o | • | o | **23** | 88 |
| 2 | 2p | o | • | o | o | o | o | o | o | o | o | o | o | o | o | • | o | o | • | o | | | • | o | o | o | **17** | 92 |
| | 2q | o | • | o | • | | | | • | • | o | o | | | • | o | | o | | o | | o | • | | o | o | **38** | 64 |
| 3 | 3p | o | o | o | • | • | o | • | o | o | o | o | o | o | o | • | o | o | o | o | o | • | | o | o | • | **26** | 92 |
| | 3q | o | o | • | • | o | o | • | o | • | o | o | o | o | o | | o | | • | o | | o | • | o | o | • | **32** | 88 |
| 4 | 4p | o | o | o | • | o | o | | • | o | | o | | o | o | • | o | o | o | o | o | • | o | o | o | o | **18** | 88 |
| | 4q | o | o | • | • | | o | • | • | • | o | o | o | o | o | o | o | o | o | o | o | • | • | o | o | • | **33** | 96 |
| 5 | 5p | o | • | | • | o | o | • | • | o | | o | o | o | o | o | o | o | o | o | | o | o | o | o | | **19** | 84 |
| | 5q | | o | | o | | o | | | | | o | | | • | • | o | o | | o | • | • | • | o | • | o | **40** | 60 |
| 6 | 6p | o | • | o | • | o | o | | • | • | | o | o | o | • | • | o | o | o | o | o | • | | o | o | o | **27** | 88 |
| | 6q | o | | o | • | o | o | | o | • | | o | | o | • | • | | o | o | | | • | • | o | o | | **35** | 68 |
| 7 | 7p | o | • | • | o | o | | | o | o | o | o | | | • | o | | o | | o | | o | o | o | o | o | **17** | 72 |
| | 7q | o | • | o | o | | o | | o | | | o | | | • | • | o | o | | | | o | o | o | o | o | **19** | 64 |
| 8 | 8p | • | | o | o | o | o | | o | o | o | o | o | | o | • | o | o | o | | | • | | o | • | • | **26** | 76 |
| | 8q | • | • | o | o | | | | • | o | o | o | | • | o | o | o | o | o | | o | o | o | | • | o | **26** | 76 |
| 9 | 9p | o | | | • | | | | | o | | | | o | • | | o | | | | o | | | o | • | o | **30** | 40 |
| | 9q | o | • | o | • | o | o | • | • | o | o | o | o | o | • | • | • | o | o | o | • | • | • | o | • | o | **48** | 100 |
| 10 | 10p | o | o | • | o | o | o | | • | o | | o | o | o | o | • | o | o | o | o | o | • | o | o | • | o | **22** | 92 |
| | 10q | o | | | o | o | | o | o | | | o | | o | • | o | • | o | o | o | | • | o | o | | | **19** | 64 |
| 11 | 11p | | • | • | | o | o | • | • | | o | o | | o | o | • | | o | | o | | • | | | | | **43** | 56 |
| | 11q | o | • | | | o | | o | • | o | o | o | o | o | o | • | o | o | | o | | • | • | o | • | o | **30** | 80 |
| 12 | 12p | o | | o | | o | o | o | | o | o | o | | | • | o | o | o | | | | o | | o | o | o | **6** | 64 |
| | 12q | | | | o | o | | • | | | o | o | o | | | o | o | o | o | o | | | • | o | o | o | **13** | 60 |
| 13 | 13q | o | • | | | | o | o | | | o | o | o | | • | o | o | o | o | o | | o | o | o | o | o | **12** | 68 |
| 14 | 14q | o | • | • | o | o | o | • | • | • | o | o | o | o | • | o | o | o | o | • | | • | • | o | • | • | **46** | 96 |
| 15 | 15q | | | o | o | o | | o | | • | • | o | o | | | • | o | | • | o | | • | • | | | o | **40** | 60 |
| 16 | 16p | | | o | o | o | o | | o | o | o | o | o | o | o | o | | | | | | | | | | | **0** | 48 |
| | 16q | o | • | o | o | o | o | o | | • | | | o | o | o | o | o | | | o | | o | o | o | o | • | **16** | 76 |
| 17 | 17p | o | • | • | • | o | | • | • | • | o | o | o | o | • | • | • | | | o | • | • | • | o | • | • | **61** | 92 |
| | 17q | o | • | • | • | o | • | • | • | • | o | o | o | o | • | • | • | o | • | o | • | • | • | | | | **64** | 88 |
| 18 | 18p | o | | o | | o | o | o | • | o | | | o | o | o | o | o | o | | o | | o | o | o | o | | **6** | 72 |
| | 18q | o | | o | o | o | | o | | • | | o | | | | | o | o | • | o | o | o | | | • | • | **27** | 60 |
| 19 | 19p | o | | • | | | | | | o | o | | | o | o | • | • | o | | | | • | | | | • | **45** | 44 |
| | 19q | o | o | • | | | o | | | | o | o | | o | • | | o | | o | | | o | o | | • | | **23** | 52 |
| 20 | 20p | | | • | • | o | o | | o | | | o | o | | o | o | o | o | | | | o | | | • | o | **21** | 56 |
| | 20q | o | o | • | • | o | o | | o | o | o | o | o | o | o | o | | o | o | | | • | o | | | • | **21** | 76 |
| 21 | 21q | o | • | o | • | o | o | o | • | • | o | o | o | o | | o | o | o | | o | | • | o | | • | • | **33** | 84 |
| 22 | 22q | o | • | • | • | o | o | o | o | o | | o | o | o | | | | o | | o | | • | o | o | | • | **28** | 72 |
| X | Xp | o | | | o | | o | | | • | | o | o | o | | • | o | o | | | | • | • | • | • | o | **40** | 60 |
| | Xq | o | | | • | o | o | o | | | o | | o | o | o | • | o | o | o | | | | | | | • | **21** | 56 |
| Tumours -> | | 6 | 65 | 41 | 53 | 3 | 3 | 42 | 54 | 41 | 8 | 0 | 0 | 3 | 44 | 50 | 15 | 3 | 22 | 4 | 31 | 61 | 50 | 4 | 50 | 41 | | |
| % loss | | | | | | | | | | | | | | | | | | | | | | | | | | | | |

Allelotyping results for all loci studied on a chromosomal arm are amalgamated to indicate the frequency with which individual arms are affected. When loss of heterozygosity is found at one locus on an arm, with retention of heterozygosity at another locus on that arm, the overall result is scored as loss of heterozygosity. The symbols used are explained in the key to Table III. The percentage of chromosomal arms affected by allele loss in individual tumours is listed at the bottom of the table.

**Figure 1** Percentage loss of heterozygosity on individual chromosomal arms.

**Figure 2** Autoradiographs of microsatellite polymorphism PCR products separated by polyacrylamide gel electrophoresis, showing examples of allele loss found in tumour no. 52. Left lane, normal lymphocyte DNA; right lane, tumour DNA; R, retention of heterozygosity; L, loss of heterozygosity.

study examination of only two loci on both 17p and 17q detected rates of allele loss for both chromosome arms which were almost identical to those expected from previous studies (Okamoto *et al.*, 1991; Eccles *et al.*, 1992; Gallion *et al.*, 1992; Jacobs *et al.*, 1993). These observations support the validity of using a small number of MSPs per chromosomal arm. Optimum density of MSPs should take into account the relative sizes of the chromosomes, but compromises are forced by the limited number of accurately localised highly informative probes for some arms. In this study efforts were made to achieve even coverage of the genome with the materials available. With the rapid expansion in numbers of MSPs, even greater probe density is now feasible.

In the tumours studied, a considerable level of genetic damage was evident, particularly affecting 5q, 9q, 11p, 14q, 15q, 17p, 17q, 19p and Xp. The high rate of allele loss for 17p and 17q is in keeping with results from previous studies (Okamoto *et al.*, 1991; Eccles *et al.*, 1992; Gallion *et al.*, 1992; Jacobs *et al.*, 1993). Similar frequencies of allele loss to those observed here have been reported for 11p (Zheng *et al.*, 1991; Gallion *et al.*, 1992; Viel *et al.*, 1992) and Xp (Yang-Feng *et al.*, 1992) in ovarian cancer, for 5q in colon cancer (Solomon *et al.*, 1987) and for 9q in urothelial cancer (Tsai *et al.*, 1990). Rearrangement of the short arm of chromosome 19 has been consistently observed in ovarian cancer (Pejovic *et al.*, 1989). Until recently, the long arms of chromosomes 14 and 15 have only been the subject of a limited examination (Sato *et al.*, 1991) in ovarian cancer, which did not detect frequent allele loss. However, a more extensive RFLP-allelotyping study (Cliby *et al.*, 1993) has cast more light on all the areas mentioned above, with 14q and 15q allele loss being found in 47% and 36% of tumours respectively. Overall, considerable similarities are evident when the results from the present study and the allelotyping study based on RFLP analysis are compared (Figure 4). The discrepancies observed may result from the relatively small numbers of tumours studied or the inclusion of low-grade tumours in the RFLP study, or may be due to differences in the distribution of the probes employed. This last possibility is unlikely, since the regions of the chromosomes examined in the instances where greatest differences were evident were common to both studies.

**Figure 3** Microsatellite mutations observed in two tumours, one at the D10S173 locus and one at the D16S265 locus. N, normal lymphocyte DNA; T, tumour DNA; ai, allelle 1; aii, allele 2; M, mutant alleles.

Although this study was not performed with the intention of achieving genotypic–phenotypic correlations, the genetic abnormalities revealed are likely to prove clinically relevant. Firstly, the high frequency of allele loss affecting the long arms of chromosomes 9 and 14 is a new finding in ovarian cancer, and strongly suggests that these are the sites of as yet uncharacterised tumour-suppressor genes. This supposition is supported by the recent observation of frequent 9q deletion in urothelial malignancy (Tsai *et al.*, 1990) and lymphoma (Offit *et al.*, 1993). The high incidence of partial loss of 9q in the tumours in this study permits initial localisation of a smallest region of the overlap of the deletions (R.J. Osborne *et al.*, in preparation).

Secondly, a surprisingly high overall prevalence of LOH was observed, with 29/41 arms showing deletion in more than 20% of informative tumours (mean percentage LOH = 28%). Similar results (mean percentage LOH = 35%) have been reported recently (Cliby *et al.*, 1993) in ovarian cancer. The high rate of allele loss in this disease contrasts with that reported in endometrial cancer ($<$10%) (Fujino *et al.*, 1993), suggesting that tumours derived from different tissues, which presumably have different pathogenesis, differ in the extent of genetic damage which accumulates during tumour progression.

Finally, this study reveals that microsatellite mutations (Aaltonen *et al.*, 1993) are very rare in ovarian cancer. Only two mutations were observed in 25 tumours examined with 68 MSPs (total 1,700 experiments). This finding distinguishes ovarian cancer from colon cancer in terms of the genetic

**Figure 4** Comparison of allele loss frequencies observed on individual chromosomal arms in the present study (■) and in a previous RFLP-based allelotyping study (□) (Cliby *et al.*, 1993).

lesions involved in tumorigenesis, since 28% of sporadic colon tumours showed microsatellite instability in a recent study (Thibodeau *et al.*, 1993). The possibility that genetic dysfunction leading to microsatellite mutation is involved in some forms of hereditary ovarian cancer has not yet been explored, since the tumours studied here were all derived from sporadic cases.

The use of PCR allelotyping to detect the multiple deletions which represent dysfunction of tumour-suppressor genes is applicable to all tumour types (assuming tissue free from excessive normal cell contamination can be obtained). Analysis of a representative panel of tumours with well-characterised clinical or pathological features will permit correlations between genetic and phenotypic parameters which are more wide-ranging and complete than those based on examination of a very limited number of genetic lesions in tumours, as was previously done. Detailed studies of tumour progression, using very small amounts of microdissected tissue or archival (formalin fixed, wax embedded) material (Greer *et al.*, 1991), are possible with this technique. Examination of epithelium from benign cysts and borderline tumours which sometimes occur synchronously with frankly malignant ovarian neoplasms will greatly clarify understanding of the pathogenesis of ovarian cancer. Concurrent examination of malignant epithelium and underlying stroma will be similarly important.

Although PCR allelotyping is capable to revealing losses of genetic material in tumours, it is unsuitable for detection of gene amplifications and thus the technique may not provide a full picture of the genetic disturbances in a particular tumour. It is also unable to detect point mutations or rearrangements, and small deletions may also be missed. The newly developed technique of comparative genomic hybridisation (CGH) (Kallioniemi *et al.*, 1992) is capable of detecting both amplification and deletion of genetic material affecting any part of any chromosome. Although this approach therefore offers some advantages over PCR allelotyping, the two methods are probably complementary. PCR allelotyping provides information about microsatellite instability and, if necessary, can be applied to map sites of interest identified by CGH, using increased numbers of MSPs.

In conclusion, we have compiled and validated a set of MSPs for detecting deletions on all chromosomes in a simple and rapid fashion. Use of this approach will not only increase understanding of the relationship between genetic lesions and clinical behaviour for particular tumour types, but will also reveal similarities and differences between neoplasms derived from histologically distinct tissues.

We would like to thank Dr Pamela Rabbitts for continued guidance. Dr Osborne is supported by the Cancer Research Campaign. We are grateful to Professor N.M. Bleehen and the University of Cambridge Clinical Oncology Research Fund for additional financial support. The authors gratefully acknowledge the histopathology expertise of Miss Beverly Wilson (Addenbrooke's Hospital Department of Pathology), statistical advice provided by Dr Davina Honess, the provision of numerous oligonucleotides by the UK HGMP Resource Centre, Harrow, and the assistance of all colleagues who participated in tumour collection.

### References

AALTONEN, L.A., PELTOMAKI, P., LEACH, F.S., SISTONEN, P., PYLKKANEN, L., MECKLIN, J.-K., JARVINEN, H., POWELL, S.M., JEN, J., HAMILTON, S.R., PETERSEN, G.M., KINZLER, K.W., VOGELSTEIN, B. & DE LA CHAPELLE, A. (1993). Clues to the pathogenesis of familial colorectal cancer. *Science*, **260**, 812–816.

CAWKWELL, L., BELL, S.M., LEWIS, F.A., DIXON, M.F., TAYLOR, G.R. & QUIRKE, P. (1993). Rapid detection of allele loss in colorectal tumours using microsatellites and fluorescent DNA technology. *Br. J. Cancer*, **67**, 1262–1267.

CLIBY, W., RITLAND, S., HARTMANN, L., DODSON, M., HALLING, K.C., KEENEY, G., PODRATZ, K.C. & JENKINS, R.B. (1993). Human epithelial ovarian cancer allelotype. *Cancer Res.*, **53**, 2393–2398.

ECCLES, D.M., RUSSELL, S.E.H., HAITES, N.E. & THE ABE OVARIAN CANCER GENETICS GROUP (1992). Early loss of heterozygosity on 17q in ovarian cancer. *Oncogene*, **7**, 2069–2072.

EHLEN, T. & DUBEAU, L. (1990). Loss of heterozygosity on chromosomal segments 3p, 6q and 11p in human ovarian carcinomas. *Oncogene*, **5**, 219–223.

FOULKES, W.D., RAGOUSSIS, J., STAMP, G.W.H., ALLAN, G.J. & TROWSDALE, J. (1993*a*). Frequent loss of heterozygosity on chromosome 6 in ovarian carcinoma. *Br. J. Cancer*, **67**, 551–559.

FOULKES, W.D., CAMPBELL, I.G., STAMP, G.W.H. & TROWSDALE, J. (1993*b*). Loss of heterozygosity and amplification on chromosome 11q in human ovarian cancer. *Br. J. Cancer*, **67**, 268–273.

FUJINO, T., RISINGER, J.I., NICHII, H., COLLINS, N.K., LIU, F.-S., CHOI, C., TAKAHASHI, H., SASAKI, H., KOHLER, M., BERCHUCK, A., BARRETT, J.C. & BOYD, J. (1993). Allelotype of endometrial carcinoma. *Proc. Am. Assoc. Cancer Res.*, **34**, 540.

GALLION, H.H., POWELL, D.E., MORROW, J.K., PIERETTI, M., CASE, E., TURKER, M.S., DEPRIEST, P.D., HUNTER, J.E. & VAN NAGELL, J.R. (1992). Molecular genetic changes in human epithelial ovarian malignancies. *Gynecol. Oncol.*, **47**, 137–142.

GANLY, P.S., JARAD, N., RUDD, R.M. & RABBITTS, P.H. (1992). PCR-based RFLP analysis allows genotyping of the short arm of chromosome 3 in small biopsies from patients with lung cancer. *Genomics*, **12**, 221–228.

GREER, C.E., PETERSON, S.L., KIVIAT, N.B. & MANOS, M.M. (1991). PCR amplification from paraffin-embedded tissues. *Am. J. Clin. Pathol.*, **95**, 117–124.

HIGUCHI, R. (1989). Simple and rapid preparation of samples for PCR. In *PCR Technology*, Erlich, H.A. (ed.) pp. 31–38. Stockton Press: New York.

JACOBS, I.J., SMITH, S.A., WISEMAN, R.W., FUTREAL, P.A., HARRINGTON, T., OSBORNE, R.J., LEECH, V., MOLYNEUX, A., BERCHUCK, A., PONDER, B.A.J. & BAST, R.C. (1993). A deletion unit on chromosome 17q in epithelial ovarian tumours distal to the familial breast/ovarian cancer locus. *Cancer Res.*, **53**, 1218–1221.

JONES, M.H. & NAKAMURA, Y. (1992). Deletion mapping of chromosome 3p in female genital tract malignancies using microsatellite polymorphisms. *Oncogene*, **7**, 1631–1634.

KALLIONIEMI, A., KALLIONIEMI, O.-P., SUDAR, D., RUTOVITZ, D., GRAY, J.W., WALDMAN, F. & PINKEL, D. (1992). Comparative genomic hybridisation for molecular cytogenic analysis of solid tumours. *Science*, **258**, 818–821.

LITT, M. (1991). PCR of TG microsatallites. In *PCR: A Practical Approach*, McPherson, M.J., Quirke, P. & Taylor, G.R. (eds) pp 85–99. Oxford University Press, New York.

OFFIT, K., PARSA, N.Z., JHANWAR, S.C., FILIPPA, D., WACHTEL, M. & CHAGANTI, R.S.K. (1993). Clusters of chromosome 9 aberrations are associated with clinico-pathologic subsets of non-Hodgkin's lymphoma. *Genes Chrom. Cancer*, **7**, 1–7.

OKAMOTO, A., SAMESHIMA, Y., YOKOYAMA, S., TERASHIMA, Y., SUGIMURA, T., TERADA, M. & YOKOTA, J. (1991). Frequent alleleic losses and mutations of the p53 gene in human ovarian cancer. *Cancer Res.*, **51**, 5171–5176.

OSBORNE, R., LEECH, V., GANLY, P., MOLYNEUX, A. & RABBITTS, P. (1992). Chromosome 3p deletion in epithelial ovarian tumours. *Proc. Am. Assoc. Cancer Res.*, **33**, 384.

PEJOVIC, T., HEIM, S., MANDAHL, N., ELMFORS, B., FLODERUS, U.-M., FURGYIK, S., HELM, G., WILLEN, H. & MITELMAN, F. (1989). Consistent occurrence of a 19p+ marker chromosome and loss of 11p material in ovarian seropapillary cystadenocarcinomas. *Genes Chrom. Cancer*, **1**, 167–171.

SAITO, S., SAITO, H., KIO, S., SAGAE, S., KUDO, R., SAITO, J., NODA, K. & NAKAMURA, Y. (1992). Fine-scale deletion mapping of the distal long arm of chromosome 6 in 70 human ovarian cancers. *Cancer Res.*, **52**, 5812–5817.

SATO, T., SAITO, H., MORITA, R., KOI, S., LEE, J.H. & NAKAMURA, Y. (1991). Allelotype of human ovarian cancer. *Cancer Res.*, **51**, 5118–5122.

SOLOMON, E., VOSS, R., HALL, V., BODMER, W.F., JASS, J.R., JEFFREYS, A.J., LUCIBELLO, F.C., PATEL, I. & RIDER, S.H. (1987). Chromosome 5 allele loss in human colorectal carcinomas. *Nature*, **328**, 616–619.

THIBODEAU, S.N., BREN, G. & SCHAID, D. (1993). Microsatellite instability in cancer of the proximal colon. *Science*, **260**, 816–819.

TODD, J.A. (1992). La carte des microsatellites est arrivee! *Hum. Mol. Genet.*, **1**, 663–666.

TSAI, Y.C., NICHOLS, P.W., HITI, A.L., WILLIAMS, Z., SKINNER, D.G. & JONES, P.A. (1990). Allelic losses of chromosomes 9, 11 and 17 in human bladder cancer. *Cancer Res.*, **50**, 44–47.

VIEL, A., GIANNINI, F., TUMIOTTO, L., SOPRACORDEVOLE, F., VISENTIN, M.C. & BOIOCCHI, M. (1992). Chromosomal localisation of two putative 11p oncosuppressor genes involved in human ovarian tumours. *Br. J. Cancer*, **66**, 1030–1036.

VOGELSTEIN, B., FEARON, E.R., HAMILTON, S.R., KERN, S.E., PREISINGER, A.C., LEPPERT, M., NAKAMURA, Y., WHITE, R., SMITS, A.M.M. & BOS, J.L. (1988). Genetic alterations during colorectal tumor development. *N. Engl. J. Med.*, **319**, 525–532.

WHANG-PENG, J., KNUTSEN, T., DOUGLASS, E.C., CHU, E., OZOLS, R.F., HOGAN, W.M. & YOUNG, R.C. (1984). Cytogenetic studies in ovarian cancer. *Cancer Genet. Cytogenet.*, **11**, 91–106.

YANG-FENG, T.L., LI, S., HAN, H. & SCHWARTZ, P.E. (1992). Frequent loss of heterozygosity on chromosomes Xp and 13q in human ovarian cancer. *Int. J. Cancer*, **52**, 575–580.

ZHENG, J., ROBINSON, W.R., EHLEN, T., YU, M.C. & DUBEAU, L. (1991). Distinction of low grade from high grade human ovarian carcinomas on the basis of losses of heterozygosity on chromosomes 3, 6 and 11 and HER-2/*neu* amplification. *Cancer Res.*, **51**, 4045–4051.


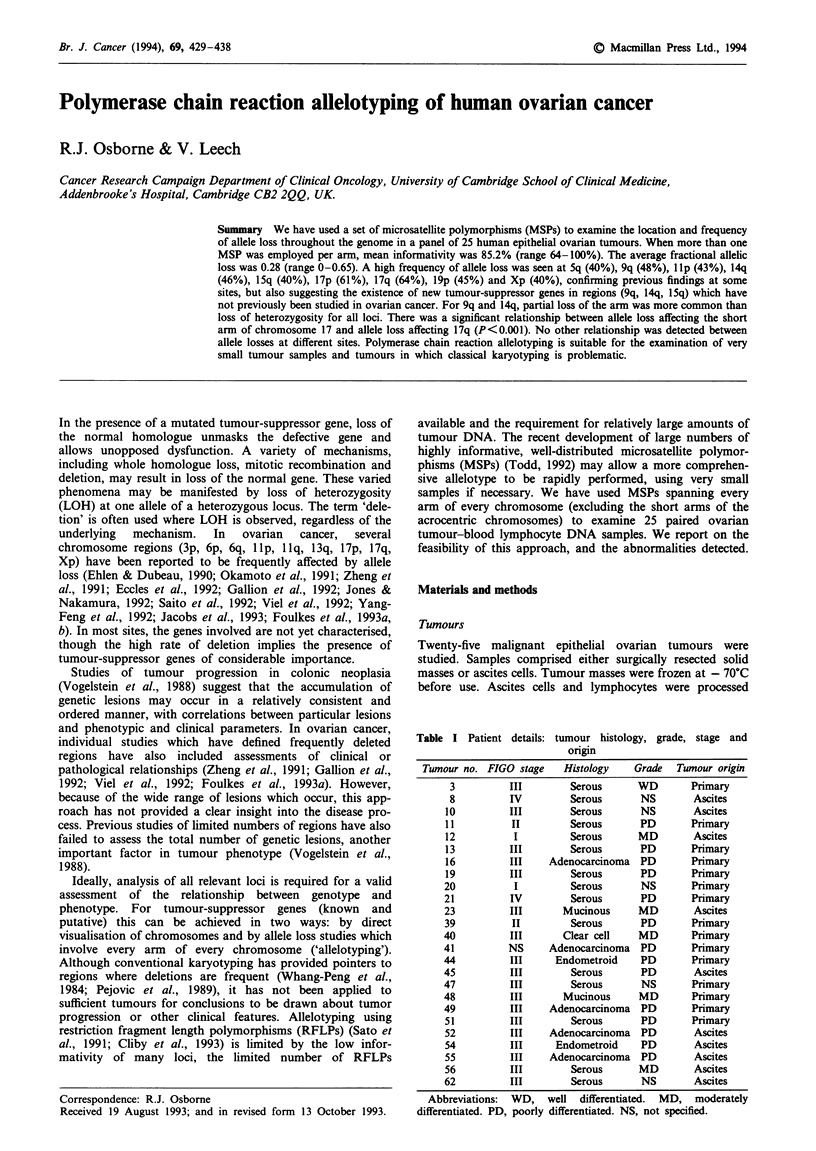

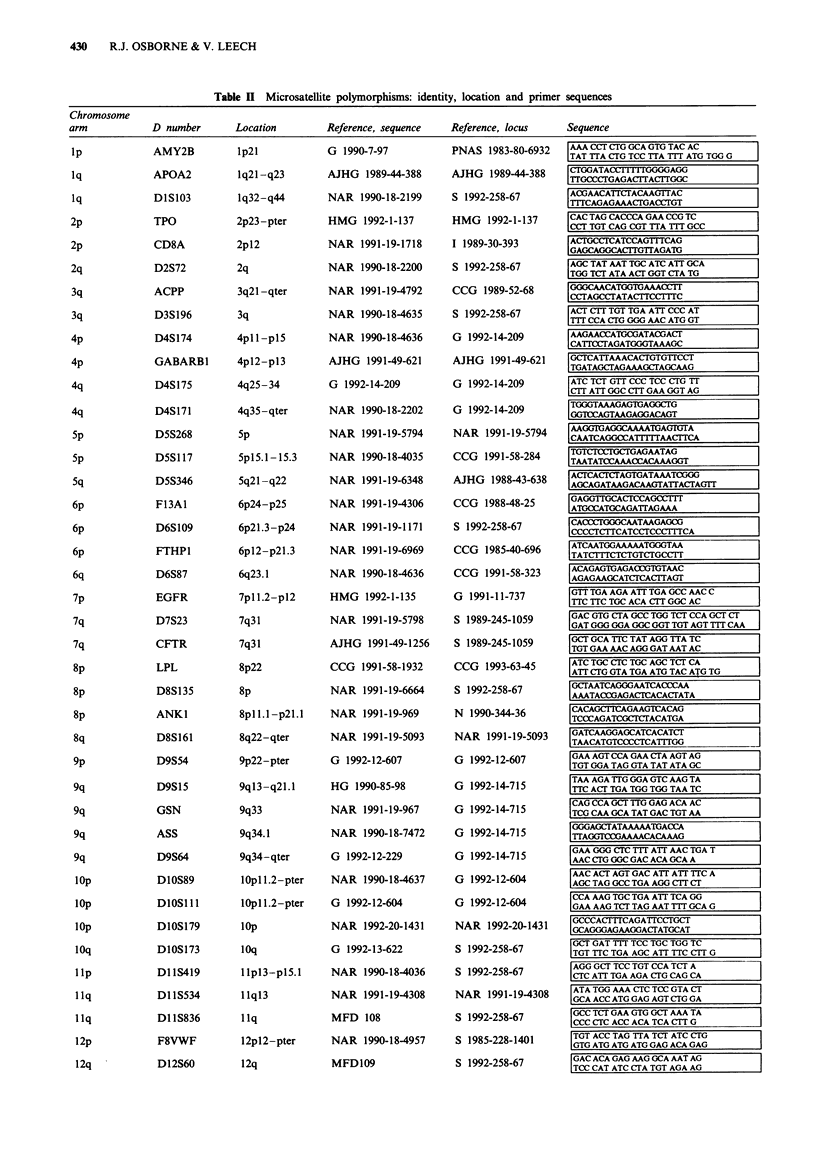

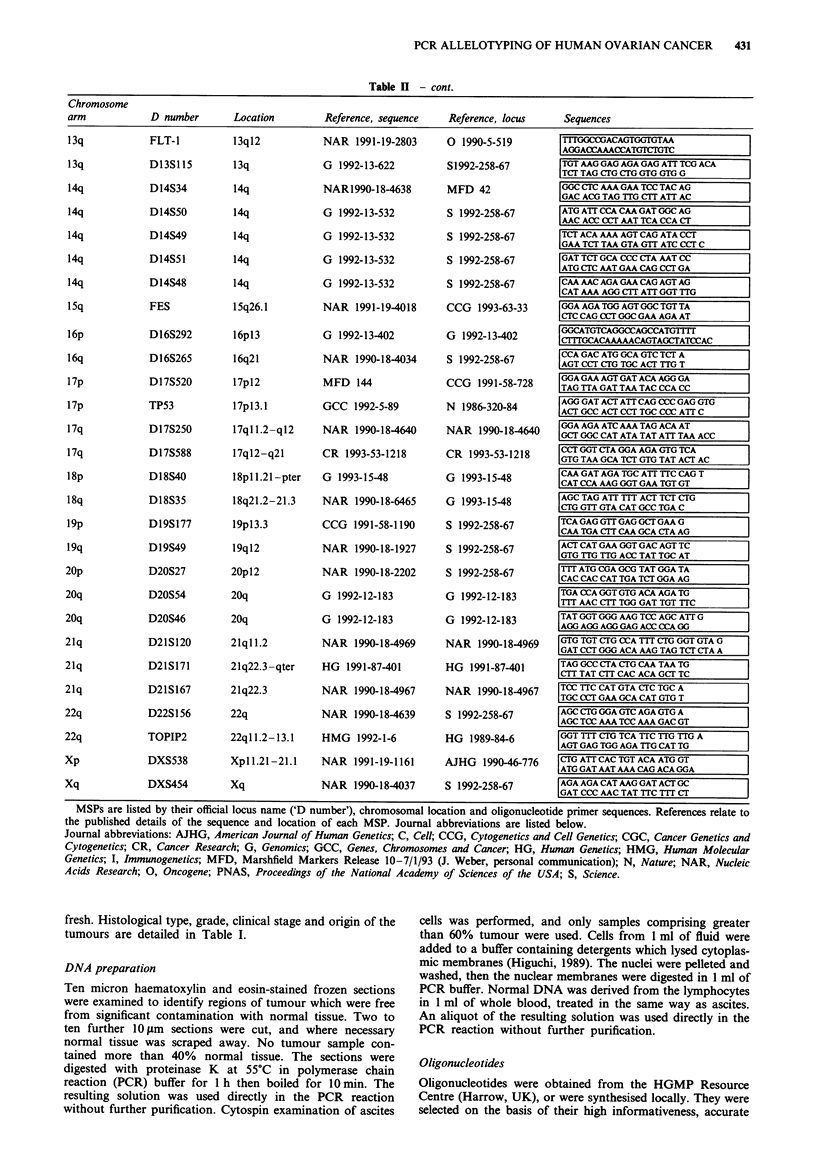

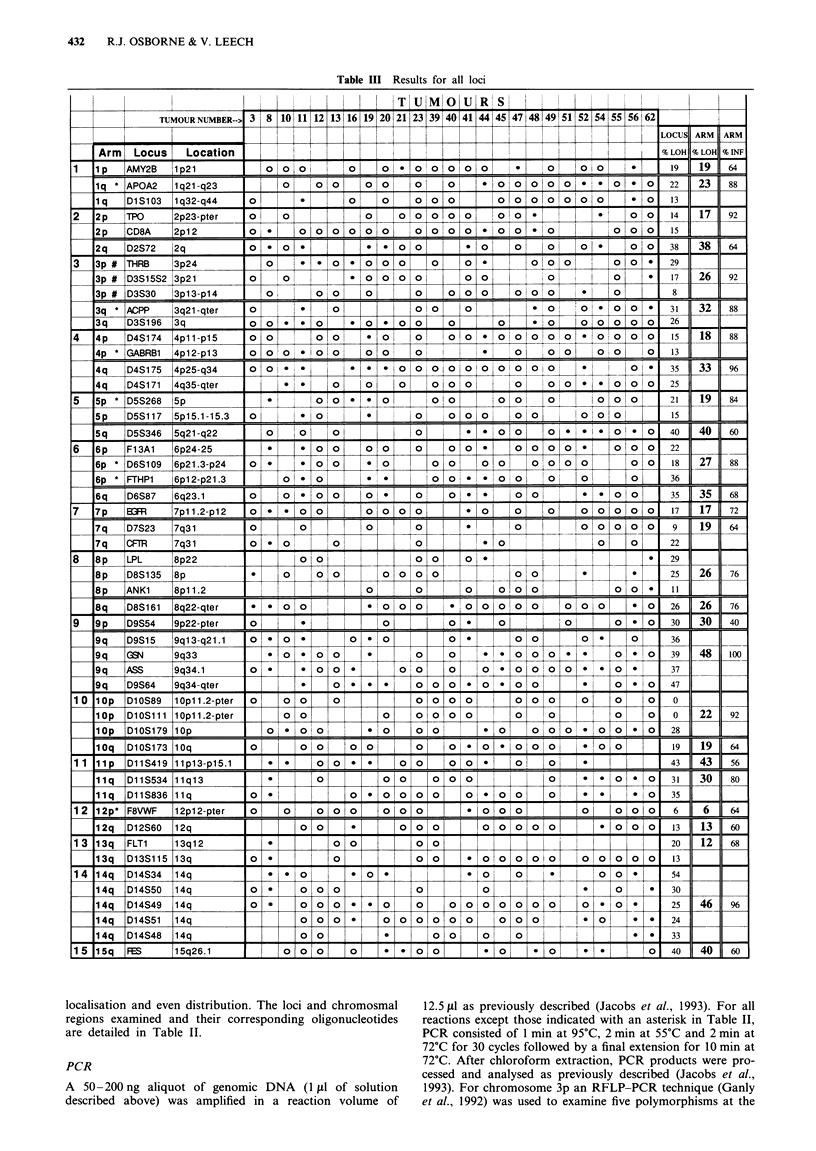

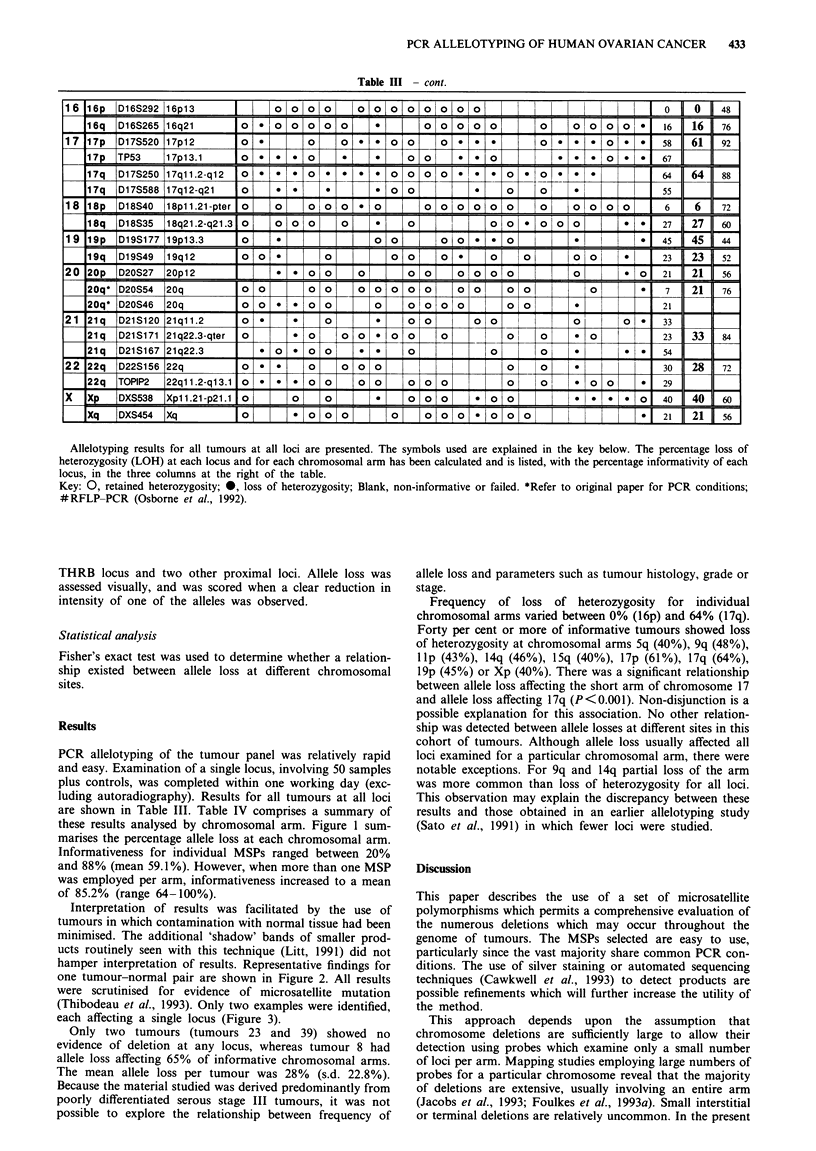

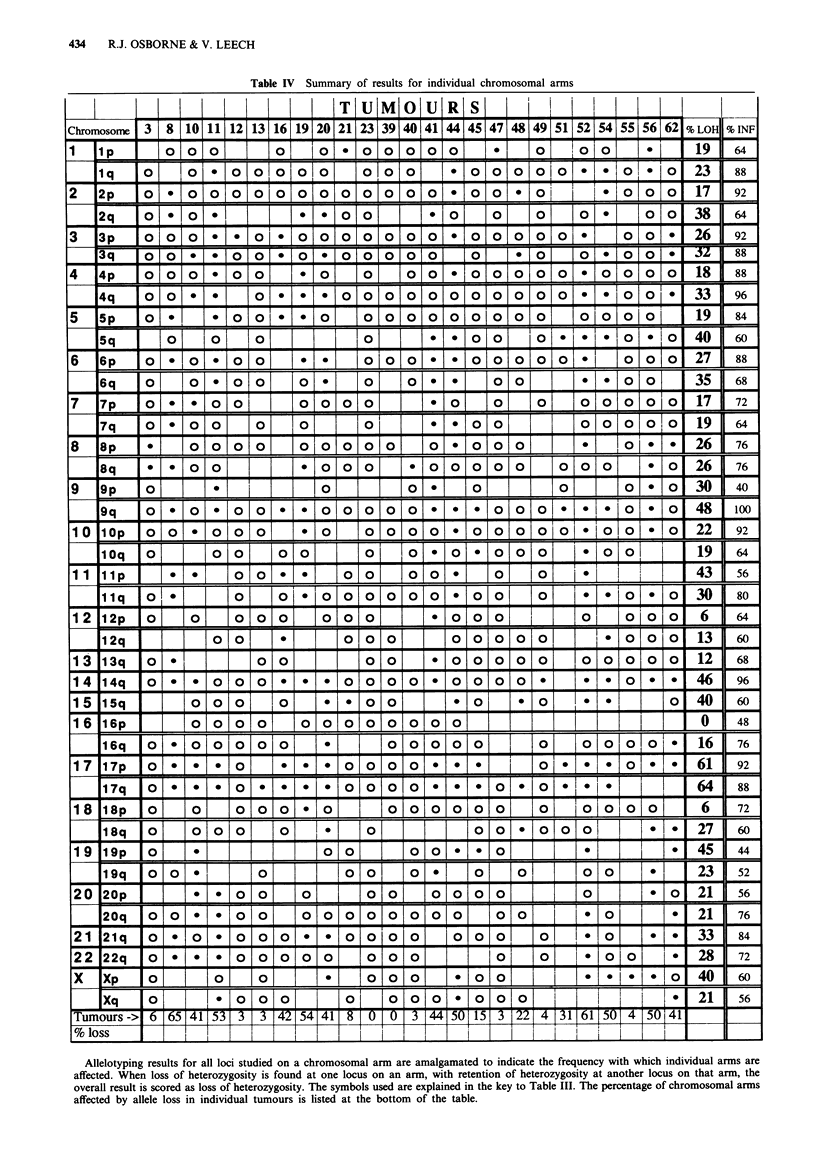

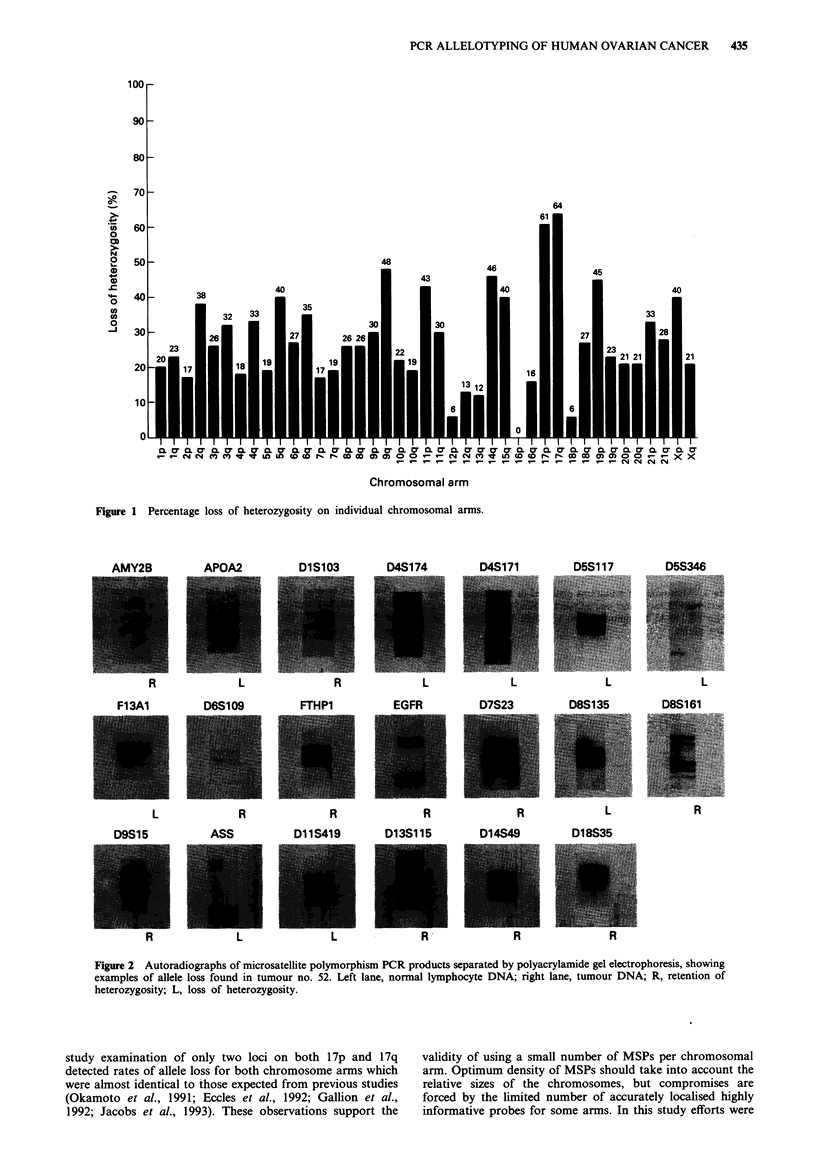

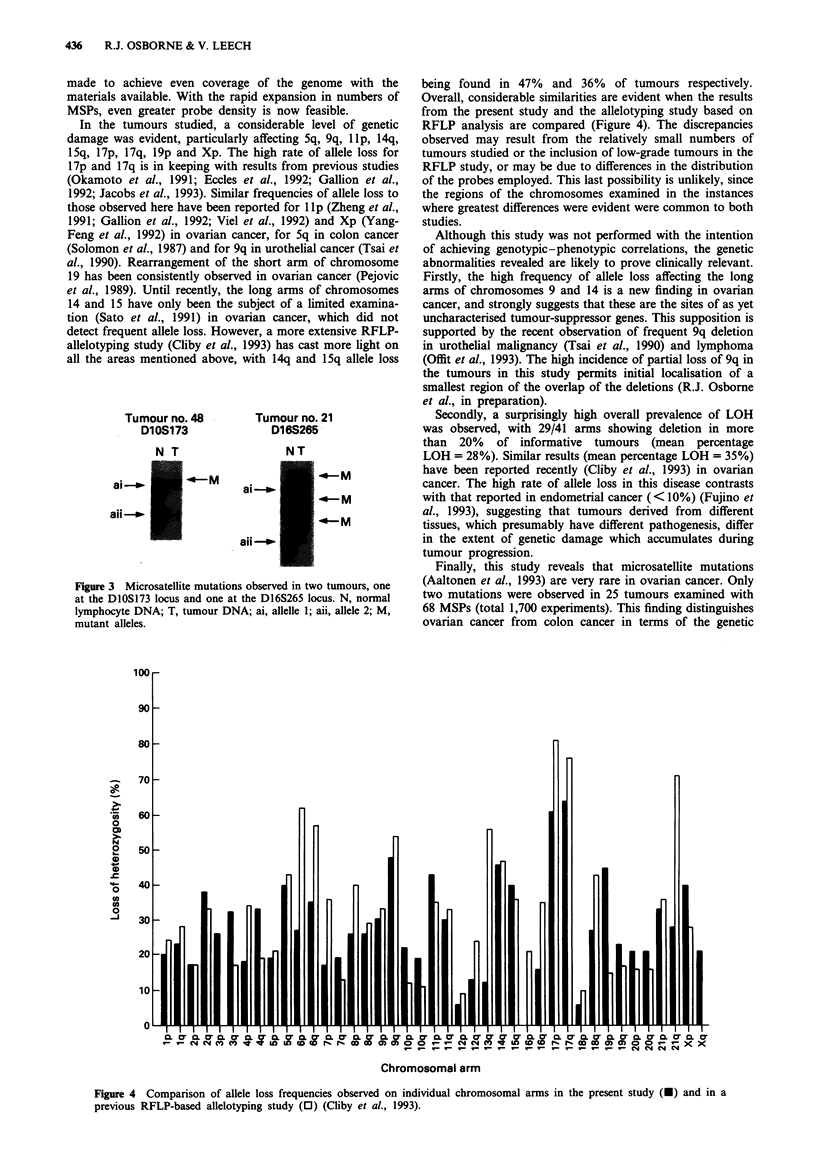

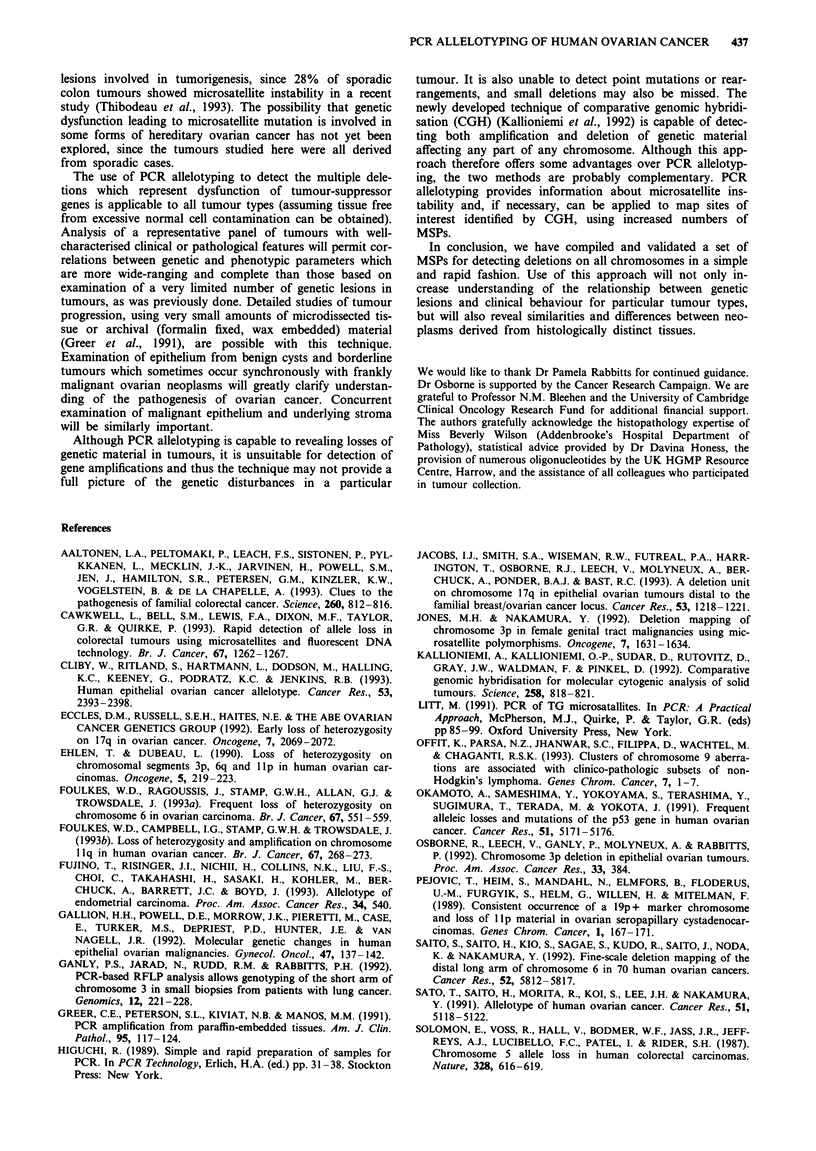

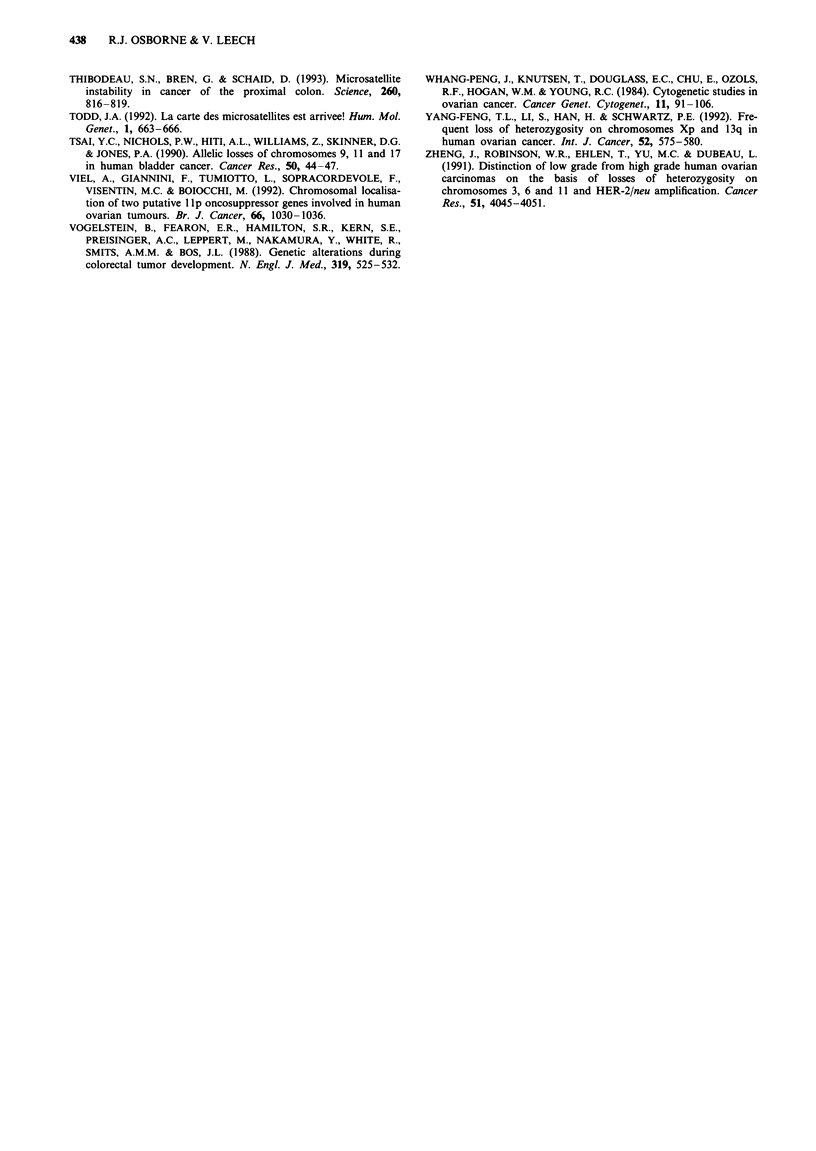


## References

[OCR_01427] Aaltonen L. A., Peltomäki P., Leach F. S., Sistonen P., Pylkkänen L., Mecklin J. P., Järvinen H., Powell S. M., Jen J., Hamilton S. R. (1993). Clues to the pathogenesis of familial colorectal cancer.. Science.

[OCR_01431] Cawkwell L., Bell S. M., Lewis F. A., Dixon M. F., Taylor G. R., Quirke P. (1993). Rapid detection of allele loss in colorectal tumours using microsatellites and fluorescent DNA technology.. Br J Cancer.

[OCR_01437] Cliby W., Ritland S., Hartmann L., Dodson M., Halling K. C., Keeney G., Podratz K. C., Jenkins R. B. (1993). Human epithelial ovarian cancer allelotype.. Cancer Res.

[OCR_01443] Eccles D. M., Russell S. E., Haites N. E., Atkinson R., Bell D. W., Gruber L., Hickey I., Kelly K., Kitchener H., Leonard R. (1992). Early loss of heterozygosity on 17q in ovarian cancer. The Abe Ovarian Cancer Genetics Group.. Oncogene.

[OCR_01448] Ehlen T., Dubeau L. (1990). Loss of heterozygosity on chromosomal segments 3p, 6q and 11p in human ovarian carcinomas.. Oncogene.

[OCR_01457] Foulkes W. D., Campbell I. G., Stamp G. W., Trowsdale J. (1993). Loss of heterozygosity and amplification on chromosome 11q in human ovarian cancer.. Br J Cancer.

[OCR_01453] Foulkes W. D., Ragoussis J., Stamp G. W., Allan G. J., Trowsdale J. (1993). Frequent loss of heterozygosity on chromosome 6 in human ovarian carcinoma.. Br J Cancer.

[OCR_01468] Gallion H. H., Powell D. E., Morrow J. K., Pieretti M., Case E., Turker M. S., DePriest P. D., Hunter J. E., van Nagell J. R. (1992). Molecular genetic changes in human epithelial ovarian malignancies.. Gynecol Oncol.

[OCR_01474] Ganly P. S., Jarad N., Rudd R. M., Rabbitts P. H. (1992). PCR-based RFLP analysis allows genotyping of the short arm of chromosome 3 in small biopsies from patients with lung cancer.. Genomics.

[OCR_01480] Greer C. E., Peterson S. L., Kiviat N. B., Manos M. M. (1991). PCR amplification from paraffin-embedded tissues. Effects of fixative and fixation time.. Am J Clin Pathol.

[OCR_01493] Jacobs I. J., Smith S. A., Wiseman R. W., Futreal P. A., Harrington T., Osborne R. J., Leech V., Molyneux A., Berchuck A., Ponder B. A. (1993). A deletion unit on chromosome 17q in epithelial ovarian tumors distal to the familial breast/ovarian cancer locus.. Cancer Res.

[OCR_01496] Jones M. H., Nakamura Y. (1992). Deletion mapping of chromosome 3p in female genital tract malignancies using microsatellite polymorphisms.. Oncogene.

[OCR_01501] Kallioniemi A., Kallioniemi O. P., Sudar D., Rutovitz D., Gray J. W., Waldman F., Pinkel D. (1992). Comparative genomic hybridization for molecular cytogenetic analysis of solid tumors.. Science.

[OCR_01512] Offit K., Parsa N. Z., Jhanwar S. C., Filippa D., Wachtel M., Chaganti R. S. (1993). Clusters of chromosome 9 aberrations are associated with clinico-pathologic subsets of non-Hodgkin's lymphoma.. Genes Chromosomes Cancer.

[OCR_01518] Okamoto A., Sameshima Y., Yokoyama S., Terashima Y., Sugimura T., Terada M., Yokota J. (1991). Frequent allelic losses and mutations of the p53 gene in human ovarian cancer.. Cancer Res.

[OCR_01529] Pejovic T., Heim S., Mandahl N., Elmfors B., Flodérus U. M., Furgyik S., Helm G., Willén H., Mitelman F. (1989). Consistent occurrence of a 19p+ marker chromosome and loss of 11p material in ovarian seropapillary cystadenocarcinomas.. Genes Chromosomes Cancer.

[OCR_01536] Saito S., Saito H., Koi S., Sagae S., Kudo R., Saito J., Noda K., Nakamura Y. (1992). Fine-scale deletion mapping of the distal long arm of chromosome 6 in 70 human ovarian cancers.. Cancer Res.

[OCR_01542] Sato T., Saito H., Morita R., Koi S., Lee J. H., Nakamura Y. (1991). Allelotype of human ovarian cancer.. Cancer Res.

[OCR_01549] Solomon E., Voss R., Hall V., Bodmer W. F., Jass J. R., Jeffreys A. J., Lucibello F. C., Patel I., Rider S. H. (1987). Chromosome 5 allele loss in human colorectal carcinomas.. Nature.

[OCR_01555] Thibodeau S. N., Bren G., Schaid D. (1993). Microsatellite instability in cancer of the proximal colon.. Science.

[OCR_01560] Todd J. A. (1992). La carte des microsatellites est arrivée! [The map of microsatellites has arrived!].. Hum Mol Genet.

[OCR_01564] Tsai Y. C., Nichols P. W., Hiti A. L., Williams Z., Skinner D. G., Jones P. A. (1990). Allelic losses of chromosomes 9, 11, and 17 in human bladder cancer.. Cancer Res.

[OCR_01569] Viel A., Giannini F., Tumiotto L., Sopracordevole F., Visentin M. C., Boiocchi M. (1992). Chromosomal localisation of two putative 11p oncosuppressor genes involved in human ovarian tumours.. Br J Cancer.

[OCR_01575] Vogelstein B., Fearon E. R., Hamilton S. R., Kern S. E., Preisinger A. C., Leppert M., Nakamura Y., White R., Smits A. M., Bos J. L. (1988). Genetic alterations during colorectal-tumor development.. N Engl J Med.

[OCR_01581] Whang-Peng J., Knutsen T., Douglass E. C., Chu E., Ozols R. F., Hogan W. M., Young R. C. (1984). Cytogenetic studies in ovarian cancer.. Cancer Genet Cytogenet.

[OCR_01586] Yang-Feng T. L., Li S., Han H., Schwartz P. E. (1992). Frequent loss of heterozygosity on chromosomes Xp and 13q in human ovarian cancer.. Int J Cancer.

[OCR_01591] Zheng J. P., Robinson W. R., Ehlen T., Yu M. C., Dubeau L. (1991). Distinction of low grade from high grade human ovarian carcinomas on the basis of losses of heterozygosity on chromosomes 3, 6, and 11 and HER-2/neu gene amplification.. Cancer Res.

